# Trace Metals in Global Air: First Results from the GAPS and GAPS Megacities Networks

**DOI:** 10.1021/acs.est.3c05733

**Published:** 2023-09-21

**Authors:** Jacob Mastin, Amandeep Saini, Jasmin K. Schuster, Tom Harner, Ewa Dabek-Zlotorzynska, Valbona Celo, Eftade O. Gaga

**Affiliations:** † Air Quality Processes Research Section, Air Quality Research Division, Environment and Climate Change Canada, Toronto, Ontario M3H 5T4, Canada; ‡ Analysis and Air Quality Section, Air Quality Research Division, Environment and Climate Change Canada, 335 River Road, Ottawa, Ontario K1A 0H3, Canada; § Faculty of Engineering, Department of Environmental Engineering, Eskişehir Technical University, 26555 Eskişehir, Türkiye

**Keywords:** ambient air, trace metals, PUF disk sampler, anthropogenic sources, GAPS network

## Abstract

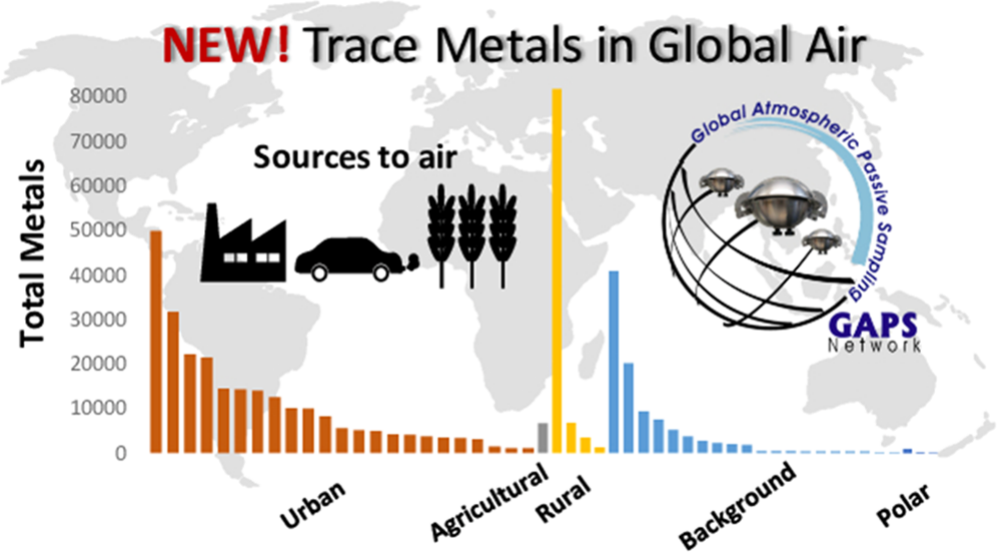

Trace metals, as constituents of ambient air, can have impacts on human and environmental health. The Global Atmospheric Passive Sampling (GAPS) and GAPS Megacities (GAPS-MC) networks investigated trace metals in the air at 51 global locations by deploying polyurethane foam disk passive air samplers (PUF–PAS) for periods of 3–12 months. Aluminum and iron exhibited the highest concentrations in air (*x̅* = 3400 and 4630 ng/m^3^, respectively), with notably elevated values at a rural site in Argentina thought to be impacted by resuspended soil. Urban sites had the highest levels of toxic Pb and Cd, with enrichment factors suggesting primarily anthropogenic influences. High levels of As at rural sites were also observed. Elevated trace metal concentrations in cities are associated with local emissions and higher PM_2.5_ and PM_10_ concentrations. Brake and tire wear-associated metals Sb, Cu, and Zn are significantly correlated and elevated at urban locations relative to those at background sites. These data demonstrate the versatility of PUF–PAS for measuring trace metals and other particle-associated pollutants in ambient air in a cost-effective and simple manner. The data presented here will serve as a global baseline for assessing future changes in ambient air associated with industrialization, urbanization, and population growth.

## Abstract 2

Low-cost passive air monitoring captures ambient trace metals at GAPS and GAPS-MC sites.

## 1 Introduction

Human exposure to air pollution increases morbidity and mortality through cardiovascular and respiratory diseases,^1^ with an estimated 4–9 million premature deaths annually attributed to air pollution exposure.^1,2^ Urban areas are associated with increased vulnerability to ambient air pollution,^3^ resulting from reliance on fossil fuels for transportation and energy generation.^1^ Particular concern has been expressed over the presence of trace metals in atmospheric particles.^4,5^ As ubiquitous components of various raw materials,^6^ anthropogenic activities such as waste incineration, fossil fuel combustion, and vehicle emissions^7−10^ are pathways for trace metals to enter the atmosphere as constituents of particulate matter (PM). Resuspension of dust and soil due to natural processes or human activities is another source of trace metals in the atmosphere.^11^ PM deposition is, among other factors, impacted by particle size, with larger particles deposited closer to their source and smaller particles subject to long-range transport.^12^ As such, the risk of human and environmental exposure to these contaminants can extend well beyond the limits of urban areas.

Due to their prevalence in the Earth’s crust and by resuspension of soil, metals such as Al, Ti, and Fe are commonly associated with coarse PM fractions (PM_2.5–10_). Other metals that are more indicative of high-temperature combustion, such as V, Ni, and Cd,^13^ are frequently observed in fine particles (≤PM_2.5_). These atmospheric fine particles pose a risk to human health, as the inhalation of PM enriched in trace metals can lead to health problems, such as oxidative stress, acute and chronic respiratory issues, and heart diseases.^14^ Research has also shown that smaller particles present increased potential for skin penetration and accumulation in the body.^15^ Some trace metals, such as Fe, Cu, Mn, and Zn, are crucial to human, animal, and plant life. However, other metals, including Ag, Cd, and Cr, are hazardous to living organisms even at low concentrations.^16−18^ Understanding that many trace elements have the potential to pose risks to both the health of humans and the well-being of natural systems, it is important to monitor the levels of these contaminants in the environment.

Polyurethane foam passive air samplers (PUF–PAS) have typically been used for the monitoring of organic pollutants in both outdoor and indoor environments.^19−22^ PUF–PAS are capable of sampling both gas- and particle-phase contaminants, including ∼PM_5_,^23^ which allows for collected samples to represent a broader mixture of chemicals present in the air. Prior studies have demonstrated the applicability of a PUF sample matrix for both the active^24,25^ and passive^26−29^ sampling of particulate-associated trace metals in the atmosphere. In comparison to active sampling methods, passive sampling has the advantage of being a more affordable alternative with lower costs associated with sample deployment and operation, including the ability to operate without an electrical source. However, there is some uncertainty associated with sampling volume estimations. Gaga et al.^28^ provided a proof of concept displaying the applicability of the Global Atmospheric Passive Sampling (GAPS)-type PUF–PAS for measuring trace metals in air. The core GAPS network, operational since 2005, has been monitoring persistent organic pollutants (POPs) and chemicals of emerging concern in global air at 111 sites, of which 40 have contributed to long-term monitoring data. Introduced in 2018, the GAPS Megacities (GAPS-MC) network has been targeting urban air pollution in 20 global megacities. For both networks, this study demonstrates the first time that inorganic contaminants were evaluated. Here, we provide an overview of trace metal concentrations through a global passive sampling network. While earlier studies have used PUF–PAS for the monitoring of trace metals, to the best of our knowledge, the current study is the first application of PUF–PAS to report trace metal concentrations on a global scale using a single monitoring network.

## 2 Materials and Methods

### 2.1 Sampling Locations

**Figure 1:**
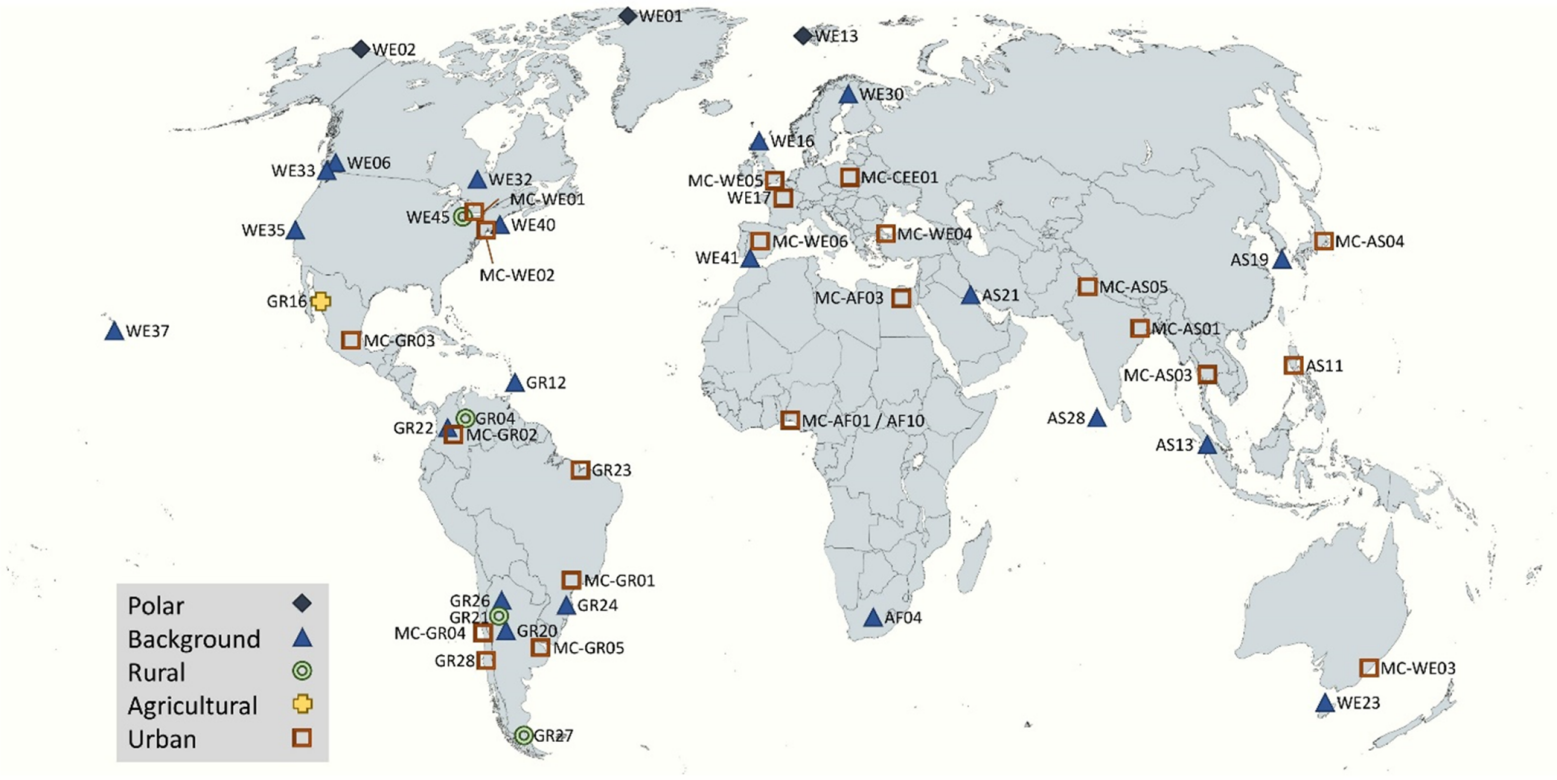
Sampling locations evaluated in the present study, encompassing sites from both the GAPS and GAPS Megacities (indicated by MC- prefix) networks. Detailed information about sampling locations, including site names, coordinates, and deployment dates, can be found in Table S1. Map source: Winkel Tripel World Map, MapChart (www.mapchart.net).

Samples were collected at 51 sites (Figure 1) between late 2018 and early 2020, representing all five United Nations (UN) regions. Sampling sites were located in urban (*n* = 23), rural (*n* = 4), agricultural (*n* = 1), background (*n* = 20), and polar (*n* = 3) locations. Of the 51 locations, 33 sites were part of the core GAPS Network,^30^ while the remaining 18 locations were GAPS-MC sites.^31^ GAPS-MC samples were deployed from July 2018 to November 2018 (∼90 days, with some exceptions), with GAPS samples deployed from January 2019 to January 2020 (∼365 days). More details about the locations and sampling periods are provided in Table S1.

### 2.2 Sampling Methods

All precleaned PUF disks (Tisch Environmental, Cleves, OH) were deployed in precleaned double-dome, stainless steel housings (TE-200-PAS; Tisch Environmental, Cleves, OH). The same sampler configuration has been used previously for the analysis of airborne organic contaminants.^21,32−34^ Field blanks (*n* = 19) were collected by exposing the PUF disk to ambient air for a few seconds, followed by storage and treatment as samples.

### 2.3 Sample Preparation, Analysis, and Quality Assurance/Quality Control (QA/QC)

Procedures for the preparation of PUF disks for the sampling of trace metals have been previously established.^28^ In the present study, a modified protocol was adopted, so the same PUF disk could be used for both organics and inorganics (trace metals) analyses. In a 4 L plastic container, 8–10 PUFs were soaked in deionized (DI) water and sonicated for 30 min. While wearing nitrile gloves, excess DI water was squeezed out. PUFs were then dried at 50 °C for 6–8 h. Using accelerated solvent extraction, PUF disks were cleaned using acetone, petroleum ether, and acetonitrile according to the method detailed in Section S1. Following this, PUF disks were rinsed 3 times with fresh deionized (DI) water. 8–10 PUFs were then sonicated in 4 L of 1% (v/v) HNO_3_ for 1.5 h. After sonication, PUF disks were rinsed multiple times in DI water to remove any remaining acid residue and then dried under N_2_. Once dry, PUF disks were transferred into precleaned amber jars using plastic forceps.

Samples were analyzed for both water-soluble and acid-digested metals at the Trent University Water Quality Center (Peterborough, Ontario, Canada). The full analytical procedure can be found in Section S2 of the Supporting Information (SI). Briefly, for the water-soluble fraction, approximately 0.25 g of PUF material was extracted in an ultrasonic bath for 30 min with 10 mL of high-purity water (18.2 MΩ). Samples were then filtered with 0.22 μm Nylon filters and acidified with 1% (v/v) HNO_3_. Acid-digested fractions were obtained from approximately 0.1 g of PUF and digested with a mixture of 7.5 mL of 40% (v/v) HNO_3_ and 2.5 mL of 30% (w/w) H_2_O_2_ at 100 °C for 24 h using a hot plate. Acid-digested extracts were diluted 10-fold before analysis. All 25 selected metals (Be, Al, Ti, V, Cr, Mn, Fe, Co, Ni, Cu, Zn, Sr, As, Se, Mo, Ag, Cd, Sn, Sb, Ba, La, Ce, Tl, Pb, U) were detected in the collected samples using an Agilent 8800 ICP-QQQ-MS, with sample introduction achieved using a MicroMist nebulizer (nominal uptake rate 400 μL/min) and a Scott double pass spray chamber. Full details of instrumentation conditions and measurement parameters are reported in Section S3. In total, 141 samples and blanks were processed (*n* = 71 for acid digestion; *n* = 70 for water extraction). The homogeneity of trace metals in wedges collected from the same PUF disk was assessed through an interlaboratory comparison, which was within 50% for 94% of coanalyzed samples (*n* = 18) (Figure S1 and SI Excel ECCC Acid-Digested).

Measured concentrations of standard reference materials were within 10% of the certified values. Matrix spike recovery was within 85–115% for most elements. Additional QA/QC details are provided in Section S4. All samples were blank-corrected. If sample concentrations were < MDL, they were substituted with 1/2 MDL. More details are provided in Section S5. Sn was omitted from further discussion due to high blank concentrations, as it has a suspected use in the PUF manufacturing processes.^24^ Data analysis was conducted in Microsoft Excel 2016 and Python. Data visualization was completed using the Matplotlib and Seaborn libraries in Python.^35,36^

### 2.4 Conversion to Concentrations in Air

Mass-based concentrations per PUF were converted into concentrations in air using site-specific sampling rates derived through the model and online tool developed by Herkert et al.^37−39^ This approach has been used in prior GAPS Network publications.^30^ Air concentration conversions were conducted as follows
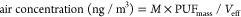
where *M* is the concentration of trace metal (ng/g PUF), PUF_mass_ is the mass of the PUF disk sampling medium (4.4 g), and *V*_eff_ (m^3^) is the effective sampling volume determined as the product of sampling duration and sampling rate (Tables S1 and S2). The resulting concentrations should be viewed as semiquantitative based on variability in sampling rates among trace metals, reported by Gaga et al.^28^

### 2.5 Determination of Enrichment Factors

To investigate the extent of the contribution of anthropogenic emissions to atmospheric elemental levels, the enrichment factor (EF) was estimated as the ratio of each element’s abundance in PM to its average abundance in the upper continental crust (UCC). Aluminum (Al) was selected as the reference element due to its high detection rates and low coefficients of variation. It is also minimally impacted by soil redox processes and biogeochemical cycling^40,41^ and exhibits little variation between UCC and bulk continental crust.^42^ As variability in reference element concentration, changing the crustal composition between regions, and mobility of trace elements through environmental compartments can all introduce uncertainty in the interpretation of EF,^43^ we minimize the error in EF by selecting a reference element that is widely detected and undergoes minimal flux in the environment. The EFs of 24 elements at all sampling locations have been calculated according to the following equation
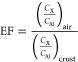
where (*C*_X_/*C*_Al_)_air_ and (*C*_X_/*C*_Al_)_crust_ are the ratios of element X to Al in air and Earth’s crust, respectively. Mason’s global compilation of soil was used as a reference soil in these calculations.^44^ Typically, elements with EFs greater than 10 are considered to derive mostly from anthropogenic sources, whereas elements with EFs approaching unity are considered mainly of crustal origin.^45^

## 3 Results and Discussion

### 3.1 Enrichment Factors

**Figure 2:**
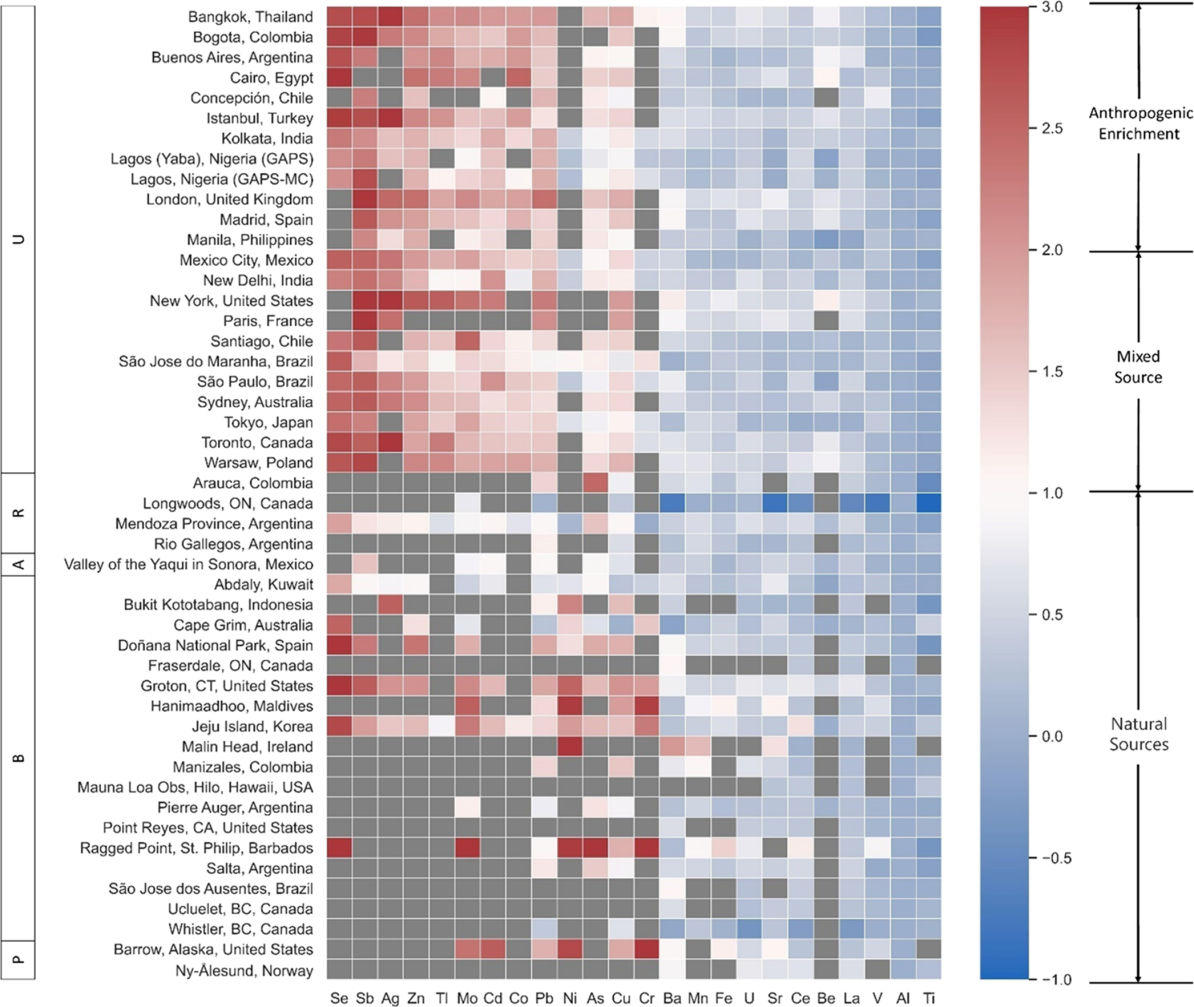
Enrichment factors (logEF) of metals at sampling locations. Locations are alphabetized and grouped by location type (U = urban, R = rural, A = agricultural, B = background, *P* = polar). Metal species sorted by the highest average enrichment. LogEF values are limited to 3, as anything above remains anthropogenically enriched. Where metal concentrations were < MDL, cells are marked in gray. De Aar (South Africa), Pallas (Finland), and Alert (Canada) have been omitted due to < MDL reference Al concentrations.

Calculated EFs are summarized in Figure 2. Full EF results for all metals are detailed in the Supporting Information (SI Excel, Enrichment Factors). High average enrichments (EFs > 100) were observed for Zn, As, Mo, Ag, Cd, and Sb, which implies that atmospheric concentrations of these elements are primarily influenced by anthropogenic sources. The average EFs for Cu and Pb were between 10 and 100, indicating that these elements originated from the mixed source of anthropogenic and crustal materials, and anthropogenic sources accounted for a considerable proportion.^45^ Other elements also exhibited EFs > 10 (e.g., Cr, Co, Ni, Se, Tl); however, the detection frequency of these metals was <50%. For all other metal species, EF values were <10, suggesting that they originated mainly from natural sources. Alert (Canada), Pallas (Finland), and De Aar (South Africa) have been omitted due to Al concentrations < MDL.

New York (USA) and Groton (USA) were enriched in most metal species (*n* = 7). Concentrations of metals in air were generally more enriched at urban sites, while those at rural sites averaged the least.

### 3.2 Total Trace Metals

**Figure 3:**
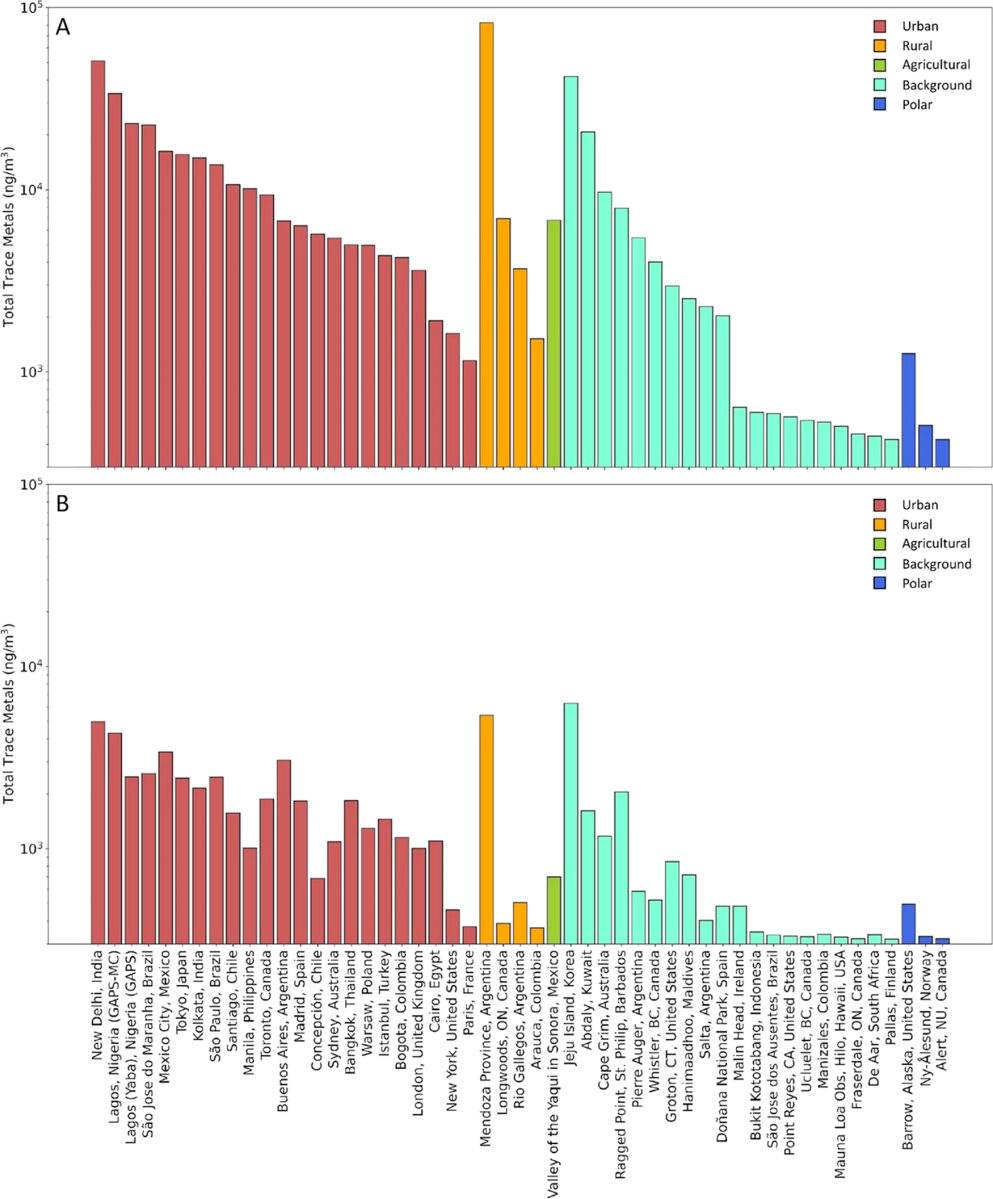
(A) Total trace metal concentrations (∑_24_Metals). (B) Total trace metal concentrations (∑_22_Metals) when Al and Fe are excluded. Sites are organized by site type and decreasing concentration.

Levels of trace metals measured at GAPS and GAPS-MC sites are summarized in Figure 3a and Supporting Information (SI Excel, Air Concentrations). For most locations, the total trace metal content, calculated as the sum of all acid-digested elements, is heavily influenced by concentrations of Al and Fe, which generally make up 50–90% of trace metals when combined. If Al and Fe are excluded, it is observed that the general trends of the total metals remain similar (Figure 3b). The highest concentrations of total trace metals (82,700 ng/m^3^) were detected in Mendoza Province (Argentina). The low EFs for most of the elements measured at this site (ranging from 0.68 to 37.0, excluding Se) suggest that the presence of metal in PM can be attributed to soil/crustal contributions from the many agricultural fields that surround the site. The next highest concentrations (51,000 ng/m^3^) were observed in New Delhi (India), where air quality is a frequent concern.^46^ With 9 elements observed having EFs > 10, of which 4 EFs were >100, there were indications of anthropogenically influenced metal concentrations. Both within and surrounding New Delhi, industry and vehicular emissions contribute substantially to poor air quality. During the sampling period in New Delhi, the air quality index for both PM_2.5_ and PM_10_ averaged approximately 150 and ranged from as low as 16 to as high as 440.^47^ The lowest total metal concentrations (425 ng/m^3^) were observed in Pallas (Finland), where most metals were < MDL. Fraserdale (Canada), Alert (Canada), Mauna Loa (United States), and De Aar (South Africa) also exhibited low levels of total trace metals. The remote nature of these sites having low PM concentrations is likely the reason for the low concentrations of trace metals. In the case of Mauna Loa, high sampling elevation may also factor into low observed levels, as trace metal concentrations generally decline with elevation.^48^ Our results for Mauna Loa are consistent with previously low levels reported at the site.^49,50^

### 3.3 Crustal Elements in Air

Crustal elements are elements that, by mass, are mainly found in coarse aerosols and include elements such as Ba, Al, Ti, Fe, Sr, La, and Ce.^51^

**Figure 4:**
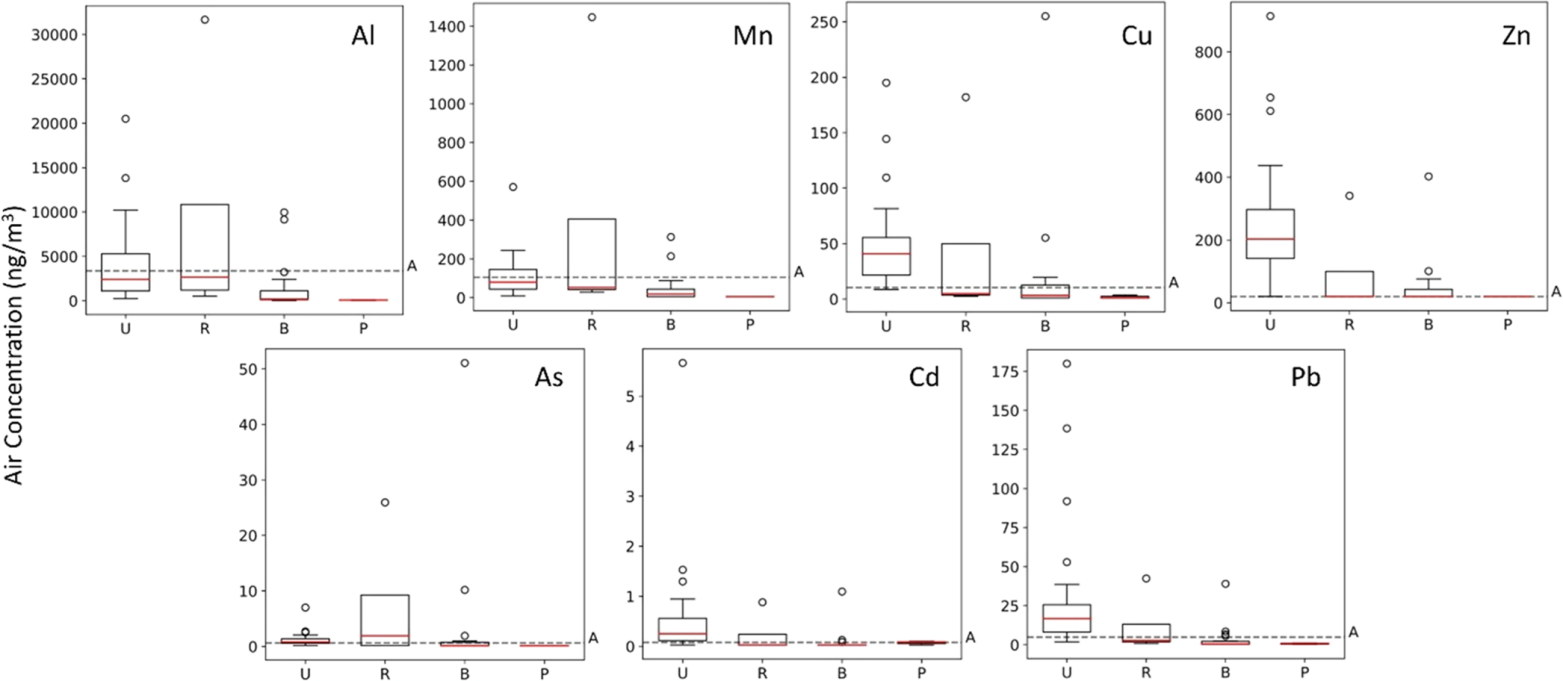
Global concentrations of Al, Mn, Cu, Zn, As, Cd, and Pb for GAPS and GAPS-MC 2018/2019 sampling year. The remaining trace metals can be found in Figures S2–S4. Boxplots illustrate the median, 25th, and 75th percentiles (whiskers marking lower/upper quartile ± IQR*1.5) and any outlying data. Concentrations are grouped by the sampling site type. Agricultural site concentrations are marked with a dashed line (site types: U = urban, B = background, R = rural, *P* = polar, A = agricultural).

Al and Fe exhibited the highest average concentrations of all metals (Table 1). This was particularly evident at rural and agricultural sites (Figures 4 and S2). Previous studies have shown a correlation between high levels of crustal metals in ambient air and proximity to agricultural fields and unpaved roads.^52^ In addition, studies of urban roadsides have suggested vehicular tires and turbulence as a resuspension mechanism of PM,^53^ which may also play a role at rural and agricultural sites where fields and unpaved roads are present. Mendoza Province (Argentina), a rural site, had the highest concentrations of both Al and Fe (31,700 ng/m^3^ and 45,600 ng/m^3^), and substantial dust deposition was visible on the PUF disk. Fraserdale (Canada) exhibited the lowest detectable Al levels (58.1 ng/m^3^). Cairo (Egypt) exhibited the lowest detectable Fe levels (425 ng/m^3^). For the urban sites investigated in this study, the average concentrations of Al and Fe were in the same order of magnitude and generally within a factor of 2.5 of values reported in other urban studies.^45,54−60^ On average, rural sites in the current study exceeded previous measurements in rural Taiwan.^61^

**Table 1:** Summary Statistics of Crustal Metals Captured by PUF–PAS at 51 Locations across the GAPS and GAPS-MC Networks during 2018/2019^a^

		Al	Ti	Fe	Sr	Ba	La	Ce
	Detection Frequency	94%	88%	76%	86%	94%	96%	96%
	1/2 MDL	28.4	1.62	77.7	0.40	0.50	0.02	0.03
Urban	Min	245	12.4	425	3.82	5.41	0.23	0.56
(*n* = 23)	Median	2420	124	2610	29.0	50.2	1.91	3.45
	Mean	4420	207	5470	35.6	76.1	3.75	8.03
	Max	20,500	1050	25,500	226	348	20.6	44.4
Rural	Min	531	9.22	620	<MDL	3.84	0.42	0.95
(*n* = 4)	Median	2660	60.6	2200	5.74	11.2	0.65	1.56
	Mean	9380	324	12700	113	119	10.1	23.5
	Max	31,700	1160	45,600	442	452	38.9	89.8
Background	Min	<MDL	<MDL	<MDL	<MDL	<MDL	<MDL	<MDL
(*n* = 20)	Median	191	9.74	561	3.51	6.01	0.14	0.31
	Mean	1520	125	2800	24.8	23.8	1.20	8.57
	Max	9960	1160	25,600	245	200	10.8	140
Polar	Min	<MDL	<MDL	<MDL	<MDL	1.21	0.05	0.11
(*n* = 3)	Median	78.6	<MDL	<MDL	2.03	4.03	0.15	0.34
	Mean	69.1	4.00	281	2.22	3.10	0.15	0.29
	Max	100	8.76	686	4.24	4.06	0.23	0.40
Agricultural	(*n* = 1)	3370	159	2730	48.8	43.3	3.00	6.45

a All units are in ng/m^3^ unless otherwise specified. For full results, please refer to the Supporting Information (SI Excel: Air Concentrations).

The concentrations of Ti were comparable between both urban and rural sites. These concentrations are on the same order of magnitude as those in past urban and rural studies (Table S3). Direct comparisons to our São Paulo, Mexico City, and Santiago measurements agree with previous studies in these cities.^56,58,62^ However, lower values have been reported elsewhere for high-volume sampling in Toronto, Buenos Aires, and Lagos.^45,63^ Concentrations in air at both background and polar sites were lower than those for urban and rural sites. No enrichment was observed at any site, as the EFs ranged from 0.09 to 2.81.

Urban and rural sites exhibited high median concentrations of Ce relative to those of background and polar sites. These higher concentrations, however, were not indicative of enrichment, as only three sites (Alert, Canada; Ragged Point, Barbados; Jeju Island, South Korea) had EFs indicating influence from both natural and anthropogenic sources.

Air concentrations of Ba and La were highest across urban sites compared with those of other site types. Sr concentrations were highest at the agricultural site. Concentrations for these metals were comparable to previously reported values in the literature (Table S3). These metals were of natural origin for nearly all sites, as suggested by their low EFs (SI Excel: Enrichment Factors).

### 3.4 Transition and Post-Transition Elements in Air

Transition and post-transition elements in this study include V, Cr, Mn, Co, Ni, Cu, Zn, Mo, Ag, Cd, Tl, and Pb.

Overall, median concentrations of Cd and Pb were highest at urban sites (Table 2). For both Cd and Pb, no concentrations were observed to exceed the Ambient Air Quality Guidelines (24 h average) set by the Ontario Ministry of the Environment, Conservation and Parks.^64^ Both metals were also found in quantities lower than reported in the previous literature (Table S3). EFs for Cd and Pb suggest that most sites experience mixed natural and anthropogenic influences, with some exceptions. Urban sites such as New York (USA) and London (UK) exhibited a high enrichment of both metals, with EFs ranging from 69.8 to 256. Fuel combustion and vehicle exhaust are prominent anthropogenic sources of Pb in the atmosphere.^65^ Cd can enter the atmosphere through coal combustion,^66^ metal industries,^67^ and vehicle exhaust.^68^

**Table 2:** Summary Statistics of Transition and Post-Transition Metals Captured by PUF–PAS at 51 Locations across the GAPS and GAPS-MC Networks during 2018/2019^a^

		V	Cr	Mn	Co	Ni	Cu	Zn	Mo	Ag	Cd	Tl	Pb
	Detection Frequency	82%	35%	78%	39%	37%	82%	57%	65%	41%	55%	41%	82%
	1/2 MDL	0.15	6.73	4.41	7.24	4.17	0.93	19.7	0.13	0.03	0.02	0.08	0.18
Urban	Min	0.75	<MDL	9.05	<MDL	<MDL	8.57	<MDL	<MDL	<MDL	<MDL	<MDL	1.82
(*n* = 23)	Median	6.72	<MDL	79.8	31	<MDL	40.8	203	1.82	0.19	0.25	0.74	16.8
	Mean	11.9	25.3	112	30	13.1	50.8	266	3.68	0.56	0.61	0.9	31.9
	Max	43.5	201	571	66.1	89.5	195	914	26.2	3.23	5.66	3.65	180
Rural	Min	1.07	<MDL	28.2	<MDL	<MDL	2.33	<MDL	<MDL	<MDL	<MDL	<MDL	0.73
(*n* = 4)	Median	2.44	<MDL	52.2	<MDL	<MDL	4.84	<MDL	0.27	<MDL	<MDL	<MDL	2.7
	Mean	17.8	14.2	395	17.4	12.7	48.5	100	1.29	0.12	0.24	0.27	12.1
	Max	65.2	36.8	1450	47.9	38.1	182	341	4.47	0.4	0.88	0.83	42.4
Background	Min	<MDL	<MDL	<MDL	<MDL	<MDL	<MDL	<MDL	<MDL	<MDL	<MDL	<MDL	<MDL
(*n* = 20)	Median	0.8	<MDL	17.8	<MDL	<MDL	3.22	<MDL	<MDL	<MDL	<MDL	<MDL	0.48
	Mean	5.15	217	47.1	9.3	91.8	20.4	51	2.56	0.04	0.09	0.1	3.48
	Max	44.6	2460	313	48.6	884	255	403	37	0.29	1.09	0.46	39.1
Polar	Min	<MDL	<MDL	<MDL	<MDL	<MDL	<MDL	<MDL	<MDL	<MDL	<MDL	<MDL	<MDL
(*n* = 3)	Median	<MDL	<MDL	<MDL	<MDL	<MDL	<MDL	<MDL	<MDL	<MDL	0.08	<MDL	0.72
	Mean	0.24	48.3	<MDL	<MDL	18.1	1.83	<MDL	0.2	<MDL	0.07	<MDL	0.66
	Max	0.42	131	<MDL	<MDL	45.8	3.63	<MDL	0.35	<MDL	0.1	<MDL	1.07
Agricultural	(*n* = 1)	6.63	<MDL	104	<MDL	<MDL	10.4	<MDL	0.46	<MDL	0.08	<MDL	4.84

a All units are ng/m^3^ unless otherwise specified. For full results, please refer to the Supporting Information (SI Excel: Air Concentrations).

Levels of V were comparable between urban and rural locations. Except for those from Toronto and New Delhi, these results were also in line with those reported in the literature (Table S3). For Toronto and New Delhi, results from the current study exceeded those of McNeill et al. and Rai et al., respectively, by nearly 50 times.^45,57^ However, the cause of this difference is unknown. Natural sources primarily influence air concentrations of V, as EFs ranged from 0.17 to 7.65 across all locations.

The concentrations of Mn at urban, rural, and background sites were all on the same order of magnitude. Urban and rural results were consistent with past Mn measurements (Table S3). In some instances where examined cities overlapped with the present study (such as Toronto (Canada), Kolkata (India), Buenos Aires (Argentina), and Lagos (Nigeria)), differences by over a magnitude were observed. Most locations exhibited little enrichment (EF = 1.01–10.2). However, atmospheric Mn in Malin Head (Ireland) appears moderately enriched, with an EF of 44.4. Given that Mn is a component of seawater,^69^ this may have influenced the observed Mn enrichment at Malin Head. However, similar Mn enrichment was not observed at other coastal locations.

Air concentrations of Mo, Cu, and Zn were highest across urban sites (Figures 4 and S3). EFs of these metals suggest that anthropogenic sources have either partially or fully contributed to their observed levels (EFs > 10). Given their known sources, it is of little surprise that these trace metals are found at high, enriched concentrations across urban regions. Mo is released through coal combustion and ore processing.^6,70^ It is also enriched in slag during copper processing.^71^ Cu is a known tracer for vehicular brake wear^65^ and is associated with fuel combustion.^72^ Tire wear^65^ and additives to engine oils^73^ are known sources of atmospheric Zn emissions. Measurements of Cu and Zn in the present study agree with those presented in previous studies, while Sb was lower than that reported in the literature (Table S3).

In the present study, Cr, Co, Ni, Ag, and Tl (Figure S3) had detection frequencies of less than 50% and will not be discussed in detail.

### 3.5 Other Trace Elements in Air

Based on the criteria used in this study, numerous trace elements are not classified as crustal, transition, or post-transition elements. This includes Be, As, Se, Sb, and U. Se had a detection frequency of less than 50% and will not be discussed in detail. Summary statistics are detailed in Tables 3 and S4. Full results for all metals are located in the Supporting Information (SI Excel: Air Concentrations).

**Table 3:** Summary Statistics of Metals Not Classified as Crustal, Transition, or Post-Transition Elements Captured by PUF–PAS at 51 Locations across the GAPS and GAPS-MC Networks during 2018/2019^a^

		Be	As	Se	Sb	U
	Detection Frequency	55%	61%	49%	57%	88%
	1/2 MDL	0.03	0.11	0.17	0.06	0
Urban	Min	<MDL	<MDL	<MDL	<MDL	0.02
(*n* = 23)	Median	0.24	0.8	0.58	2	0.13
	Mean	0.29	1.23	0.81	3.49	0.27
	Max	0.99	7.01	2.53	20.1	2
Rural	Min	<MDL	<MDL	<MDL	<MDL	0.04
(*n* = 4)	Median	<MDL	1.9	<MDL	<MDL	0.08
	Mean	0.47	7.47	0.53	0.4	0.79
	Max	1.8	26	1.6	1.42	2.96
Background	Min	<MDL	<MDL	<MDL	<MDL	<MDL
(*n* = 20)	Median	<MDL	<MDL	<MDL	<MDL	0.01
	Mean	0.06	3.39	0.48	0.22	0.08
	Max	0.35	51.1	3.89	2.24	0.55
Polar	Min	<MDL	<MDL	<MDL	<MDL	<MDL
(*n* = 3)	Median	<MDL	<MDL	<MDL	<MDL	0.01
	Mean	<MDL	<MDL	<MDL	<MDL	0.01
	Max	<MDL	<MDL	<MDL	<MDL	0.01
Agricultural	(*n* = 1)	0.15	0.61	<MDL	0.32	0.16

a All units are ng/m^3^ unless otherwise specified. For full results, please refer to the Supporting Information (SI Excel: Air Concentrations).

Rural sites exhibited the highest concentrations of As, followed by urban sites (Table 2 and Figure 4). Levels of As at Ragged Point (Barbados; 51.1 ng/m^3^) and in Mendoza Province (Argentina; 26.0 ng/m^3^) greatly exceeded those at all other locations (ranging from < MDL to 10.2 ng/m^3^). Ragged Point exceeded the WHO guidelines on ambient As concentrations (30 ng/m^3^),^74^ but both were below the Ambient Air Quality Guidelines (24 h average) set by the Ontario Ministry of the Environment, Conservation and Parks.^64^ EFs at Ragged Point (Barbados) and Arauca (Colombia) were much greater than 100, indicating that anthropogenic sources primarily influenced As concentrations at these sites. As has been used as an insecticide, herbicide, and wood preservative, with other electrical, industrial, and medical applications.^75^ As has been identified as a prominent component of particulate matter originating through the incineration of chromated copper arsenate-treated wood.^76^ It is also emitted through coal combustion^65^ and fuel exhaust.^77^

Air concentrations of Be and Sb were high across urban sites (Figures 4 and S4). Low enrichment of Be across most sites suggests that Be in the atmosphere is attributable to natural sources. EFs for Sb, however, suggest that anthropogenic sources have either partially or fully contributed to observed levels (EFs > 10). Sb has been noted as a prominent indicator of brake wear since the addition of Sb_2_S_3_ (antimony trisulfide) as a component of brake pads.^78−80^ Sb also originates through industrial activities such as mining and pharmaceutical manufacturing.^81^

Urban and rural sites had comparable levels of atmospheric U and were approximately eight times higher than that observed at background and rural sites. EFs at all locations were less than 10, indicating that detectable U in outdoor air is consistent with natural contributions.

### 3.6 Water-Soluble Trace Metals

The water solubility of trace metal PM constituents is important in understanding the bioaccessibility of these pollutants, including their toxicological behavior and reactive oxygen species (ROS) activity.^14,82,83^ Notably, the oxidative capacity of PM is primarily derived from its water-soluble trace elements.^84^

Fernández Espinosa et al. reported concentrations of four trace metal fractions in fine urban particles, finding little water solubility of Ti and Fe, with <8% of total Ti and <4% of total Fe being water-soluble.^85^ While the present study examined only two metallic fractions, our results are comparable, as water-soluble Ti and Fe averaged <6% and <7%, respectively. Water-soluble V, Ni, Co, Mn, Cu, Cd, and Pb were found by Fernández Espinosa et al. to be 50.5, 39.6, 35.1, 32.6, 26.4, 24.8, and 3.9% of their total concentrations, respectively.^85^ Water-soluble Pb and Cd in the present study was on average higher, at 12.4 and 50.3%, respectively, while Mn (32.9%) exhibited a similar ratio. Other water-soluble fractions found in this study were lower than the approximate 25–50% range shown by Fernández Espinosa et al.^85^ These elements also exhibited lower water solubility than at near-road urban sites in Canada.^86^

The highest water solubility was observed for Sr and Cd, as >50% of each metal was detected in the water-soluble fraction on average. Tl exhibited the lowest water solubility with only 4.3% in a water-soluble form.

The highest overall levels of total water-soluble metals were found in Mendoza Province (Argentina), Jeju Island (Korea), Kolkata (India), Lagos (Nigeria), and New Delhi (India). The lowest levels were found in De Aar (South Africa), Ucluelet (Canada), Mauna Loa (United States), Pallas (Finland), and Fraserdale (Canada). A more detailed analysis of these results is beyond the scope of this study. Water-soluble metal concentrations are summarized in the Supporting Information (SI Excel: Water-Soluble Concentrations).

### 3.7 Trace Metals and PM Correlation

Average PM_2.5_ and PM_10_ concentrations were derived by using available air quality index data at GAPS-MC locations (Table S4). The correlation between urban metals and PM concentrations was evaluated. Lagos (Nigeria; lack of reference PM_2.5_ and PM_10_ data) and New Delhi (India; outlying values) were omitted from this analysis. The full correlation matrix can be found in Table 4.

**Table 4:** Correlation Matrix for PM_2.5_, PM_10_, and Analyzed Trace Metals at GAPS-MC Locations Using Pearson Correlation Coefficients. Italicized values indicate *p* < 0.05; bolded values indicate *p* < 0.01

	PM_2.5_	PM_10_	Be	Al	Ti	V	Cr	Mn	Fe	Co	Ni	Cu	Zn
PM_2.5_	1												
PM_10_	**0.84**	1											
Be	0.2	–0.07	1										
Al	0.44	–0.14	0.48	1									
Ti	*0.61*	–0.03	0.44	**0.92**	1								
V	0.29	–0.15	0.41	**0.94**	**0.84**	1							
Cr	0.23	–0.17	0.35	**0.83**	**0.7**	**0.83**	1						
Mn	0.4	–0.13	0.43	**0.85**	**0.89**	**0.84**	**0.75**	1					
Fe	0.4	–0.2	0.33	**0.9**	**0.9**	**0.87**	**0.84**	**0.97**	1				
Co	0.15	0.05	0.5	*0.52*	0.33	0.42	*0.5*	0.25	0.31	1			
Ni	0.16	–0.16	0.22	**0.82**	**0.7**	**0.92**	**0.89**	**0.76**	**0.85**	0.44	1		
Cu	**0.61**	–0.17	0.37	**0.84**	**0.74**	**0.73**	**0.68**	*0.6*	**0.66**	0.36	*0.54*	1	
Zn	0.22	–0.27	0.38	**0.87**	**0.65**	**0.85**	**0.84**	*0.57*	**0.68**	*0.6*	**0.77**	**0.84**	1
Sr	0.38	–0.17	*0.61*	**0.88**	**0.72**	**0.84**	**0.74**	**0.67**	**0.71**	0.48	**0.67**	**0.86**	**0.85**
As	*0.6*	–0.02	0.43	**0.67**	**0.68**	*0.61*	0.4	*0.57*	*0.51*	0.16	0.3	**0.82**	*0.59*
Se	0.03	**0.75**	0.22	0.29	0.17	0.29	0.31	0.13	0.11	0.47	0.28	0.15	0.3
Mo	0.46	0.14	0.11	*0.5*	*0.6*	0.48	0.13	0.48	0.39	–0.04	0.2	*0.58*	0.3
Ag	–0.22	–0.28	0.47	0.15	0.0	0.09	0.3	0.13	0.09	0.04	–0.03	0.25	0.26
Cd	0.42	–0.15	0.18	**0.71**	**0.64**	*0.51*	**0.73**	*0.51*	**0.65**	*0.55*	*0.54*	**0.65**	**0.69**
Sb	0.39	–0.25	0.24	**0.78**	**0.64**	**0.65**	*0.51*	*0.52*	*0.58*	0.3	0.42	**0.91**	**0.77**
Ba	*0.58*	–0.21	0.39	**0.79**	**0.71**	*0.57*	**0.67**	*0.54*	**0.66**	0.48	0.47	**0.84**	**0.71**
La	0.42	–0.19	0.49	**0.75**	**0.71**	*0.53*	**0.64**	*0.58*	**0.66**	**0.69**	0.49	*0.59*	**0.64**
Ce	*0.55*	–0.14	0.39	**0.78**	**0.77**	*0.57*	**0.69**	**0.66**	**0.74**	0.48	*0.51*	**0.71**	**0.64**
Tl	–0.11	–0.16	**0.83**	0.47	0.32	0.4	0.41	0.41	0.32	0.44	0.22	0.38	0.45
Pb	0.51	–0.19	0.45	**0.71**	**0.76**	**0.69**	**0.74**	**0.66**	**0.72**	0.29	**0.68**	*0.61*	*0.62*
U	*0.52*	–0.17	*0.51*	**0.76**	**0.72**	*0.56*	**0.69**	**0.69**	**0.75**	0.39	0.46	**0.72**	*0.59*

	Sr	As	Se	Mo	Ag	Cd	Sb	Ba	La	Ce	Tl	Pb	U
PM_2.5_													
PM_10_													
Be													
Al													
Ti													
V													
Cr													
Mn													
Fe													
Co													
Ni													
Cu													
Zn													
Sr	1												
As	**0.71**	1											
Se	0.3	0.09	1										
Mo	0.43	**0.82**	0.08	1									
Ag	0.4	0.23	0.21	–0.07	1								
Cd	0.5	0.29	0.17	0.08	0.1	1							
Sb	**0.78**	**0.74**	0.13	**0.63**	0.27	*0.59*	1						
Ba	**0.73**	*0.52*	0.09	0.25	0.23	**0.84**	**0.76**	1					
La	0.56	0.34	0.15	0.11	0.12	**0.9**	*0.54*	**0.83**	1				
Ce	*0.61*	0.4	0.14	0.16	0.17	**0.92**	**0.64**	**0.9**	**0.92**	1			
Tl	*0.62*	0.39	0.37	0.15	**0.76**	0.22	0.41	0.33	0.42	0.34	1		
Pb	**0.68**	0.43	0.06	0.18	0.1	*0.58*	0.39	*0.6*	*0.59*	**0.64**	0.29	1	
U	**0.72**	0.49	0.12	0.2	0.41	**0.76**	**0.64**	**0.91**	**0.8**	**0.9**	0.48	*0.59*	1

Numerous significant correlations between coarse mode-associated metals (e.g., Al, Ti, Fe, Sr) were observed. This further supports the low enrichments of these metals found in the present study and suggests natural origins. Low enrichment of Ba, Ce, and U and their significant correlations with Al, Fe, and Ti (*p* < 0.01) also suggest crustal origins. Known markers of brake and tire wear Sb, Cu, and Zn displayed significant relationships with each other.^65,78,79^ These relationships, combined with the prevalence of vehicles in urban regions and the enrichments of Sb, Cu, and Zn, imply that the presence of these species in outdoor air is influenced by vehicle brake and tire wear. Indicators of fuel combustion (e.g., Cu, Pb, Ni, and Sr) demonstrated strong, significant relationships. Weaker and less significant correlations were observed for metals indicative of coal combustion (e.g., As, Be, Cd, Cr, Ag, and Tl) and vehicle exhaust (e.g., As, Be, Cd, Cr, Pb). Cities with high concentrations of both PM_2.5_ and PM_10_ (e.g., New Delhi, Santiago, Kolkata) broadly exhibited higher concentrations of trace metals than locations where PM_2.5_ and PM_10_ were less abundant. As high PM leads to high trace metal content, especially in heavily populated urban areas, this can lead to a greater toxic burden.

## 4 Limitations and Future Directions

While PUF–PAS are cost-effective and easy to deploy, they provide semiquantitative results representing a time-weighted average value. As such, variations in contaminant concentrations due to small-scale meteorological effects or seasonal influences may not be reflected. Long-term monitoring studies should ensure that consecutive sampling periods are used to better characterize seasonal differences in metal concentrations as exposure can vary from season to season. Beyond this, variability in sampling rates may also lead to some uncertainty in the results presented here, which apply a generic sampling rate. This has been discussed by Gaga et al.^28^ for trace metals and more generally in Herkert et al.^38^ where sampling rates were derived for GAPS core network sites based on depuration compounds. Harner et al.^87^ reported similarity in PUF–PAS sampling rates for a wide range of gas- and particle-associated chemicals as determined by several international groups and calibration studies. Generally, it has been reported that the average sampling rate at GAPS sites is 4 ± 2 m^3^/day.^38,87^

Further work needs to be conducted to evaluate the differences in trace metal uptake between active and passive sampler designs. Passive sampling has been shown to collect higher concentrations of metals than comparable active sampling, demonstrated both in this study and elsewhere.^29^ Intercomparison studies using colocated PUF–PAS and conventional PM_2.5_ and PM_10_ samplers would aid in better characterizing these differences. The GAPS and GAPS-MC networks will continue PUF–PAS monitoring to provide global baseline levels and to track changes in trace metal levels over time. Particular attention should be directed to metal species exhibiting enriched concentrations beyond natural levels and species where exposure has been linked to oxidative stress. Improved understanding of trace metal interactions with PM, as well as contributions from tire wear and brake wear, will have implications on urban health and may be of interest to regulatory agencies for guiding risk assessment and public policy.

## Supporting Information Available

Sample precleaning, extraction procedure, instrument operating conditions, QA/QC, and data processing; GAPS-MC comparison between S&T Laboratories and Trent University; remaining trace metal air concentration boxplots; sampling location and deployment details; and literature reported trace metal concentrations (PDF)
Spike recoveries, field blanks and MDLs, laboratory intercomparison results, enrichment factors, acid-digested air concentrations, and water-soluble air concentrations (XLSX)
