# Supplementary material for: Trace Metals in Global Air: First Results from the GAPS and GAPS Megacities Networks
Source: Environ Sci Technol. 2023 Sep 21;57(39):14661–73. doi: 10.1021/acs.est.3c05733 (PMC10552545; doi:10.1021/acs.est.3c05733)
Supplement: Supplementary file 1 — es3c05733_si_001.pdf [file es3c05733_si_001.pdf]

Supporting Information

Trace Metals in Global Air: First Results from the GAPS and GAPS Megacities Networks

Jacob Mastin[a,*], Amandeep Saini[a], Jasmin K. Schuster[a], Tom Harner[a], Ewa Dabek-Zlotorzynska[b], Valbona Celo[b], Eftade O. Gaga[c]

[a] Air Quality Processes Research Section, Air Quality Research Division, Environment and Climate Change Canada, Toronto, Ontario, M3H 5T4, Canada
[b] Analysis and Air Quality Section, Air Quality Research Division, Environment and Climate Change Canada, 335 River Road, Ottawa, Ontario, K1A 0H3, Canada
[c] Faculty of Engineering, Department of Environmental Engineering Eskişehir Technical University, 26555 Eskişehir, Turkey

*Email: jacob.mastin@ec.gc.ca

Contents

S1: Precleaning and Preparation of PUF Disks S1
S2: Water soluble and acid digested trace metals extraction S2
S3: ICP-MS analysis and instrument operating conditions S2
S4: QA/QC S2
S5: Data Processing. S2
Figure S1: GAPS-MC Comparison between S&T Laboratories and Trent University. S3
Figure S2: Ba, Ti, Fe, Sr, La, Ce Air Concentration Boxplots. S4
Figure S3: Cr, Co, Se, Ni Air Concentration Boxplots. S5
Figure S4: Be, V, Mo, Sb, Ag, Tl, U Air Concentration Boxplots S5
Table S1: Sampling location and deployment details S6
Table S2: Site specific sampling rates and total volume sampled S8
Table S3: Literature reported trace metal concentrations S10
Table S4: Average $PM_{2.5}$ and PM_{10} concentrations at GAPS-MC sites S12
References S13

S1 Precleaning and Preparation of PUF Disks

In a 4L plastic container, 8-10 PUFs were soaked with deionized (DI) water and sonicated for 30 minutes. While wearing nitrile gloves, excess DI water was squeezed out. PUFs were then dried at 50 ^oC for 6-8 hours. Metal ASE cell components were baked at 125 ^oC overnight prior to use. All ASE cell components were rinsed 3 times with Acetone (ACE), Ethyl Acetate, and Petroleum Ether (PE) prior to cell assembly. After PUFs were added to the fully assembled cells, they were rinsed using the following procedures in sequence (ACN=Acetonitrile):

Media	ASE Method	Solvent	Ratio	Number of extractions	Cycles	Temp (^oC)	Static	Flush %	Purge
Cleaning									
**PUF**	4	100% ACE	1	1	1	75	5	100	240
	5	75:25 PE:ACE	3:1	1	1	75	5	100	240
	6	85:15 PE:ACE	5:1	1	1	75	5	100	240

	7	100% ACN	1	1	1	50	5	100	240

Following this, PUF disks were rinsed 3 times with fresh deionized (DI) water. 8-10 PUFs were then sonicated in 4L of 0.1% (v/v) HNO_3 for 1.5 hr. After sonication, PUF disks were rinsed multiple times in DI water to remove any remaining acid residue, and then dried under N_2. Once dry, PUFs disks were transferred into pre-cleaned amber jars using plastic forceps.

S2 Water soluble and acid digested trace metals extraction

The water-soluble fraction utilized approximately 0.25 g of PUF material. This was extracted with 10 mL of high purity water (18.2 MΩ) using an ultrasonic bath for 30 min, and then filtered using 0.22 µm Nylon syringe filters. Extracted water-soluble solutions were acidified with 1% (v/v) HNO_3 prior to ICP-MS analysis. The acid-soluble fraction utilized approximately 100 mg of PUF material. PUF material was transferred into 30 mL PFA vials, and digested with a mixture of 7.5 mL of 40% (v/v) HNO_3 and 2.5 mL of H_2O_2 for 24 hours using a hotplate at 100^oC. Extracted acid-soluble solutions were diluted 10-fold prior to ICP-MS analysis.

S3 ICP-MS analysis and instrument operating conditions

Samples were analyzed using an Agilent 8800 ICP-QQQ-MS, at the Water Quality Center at Trent University (Peterborough, Ontario, Canada). A MicroMist nebulizer (nominal uptake rate 400 μL min^{-1}) and Scott double pass spray changer were used for sample introduction. Instrument operating conditions and measurement parameters are provided below:

RF power	1550 W
Carrier gas flow rate*	1.05 L min^{-1}
Sampling depth*	10 mm
Extraction lens	X-type
Sampler and skimmer cone	Standard nickel

*General Purpose Plasma Preset

Aluminum and the transition metals were measured in single quad mode with a He cell gas. Hydrogen gas and MS/MS scan mode was used for the measurement of Fe and Se. Oxygen reaction gas and MS/MS scan mode was used for the measurement of As, La, and Ce. All other elements were measured in no gas mode.

Matrix-matched calibration standards were prepared fresh daily by serial dilution of a 1000 ng mL^{-1} multielement standard. Lithium-6, Ge, Rh, and Bi were used as internal standards (~200 ng mL^{-1}), added online using a tee. For the acid-soluble fraction, 10% isopropyl alcohol was added to the internal standard solution for carbon buffering, to ensure the accurate quantification of As and Se).

For Pb, the sum of the three major isotopes (^{206}Pb, ^{207}Pb, and ^{208}Pb) was used to account for any isotopic variability in the samples (due to their geographic origin).

S4 QA/QC

NIST SRM 1640a (Trace Elements in Natural Water), CALA PT sample (CO2-1 Full Range Metals, October 2021 Study), and USGS rock standard BCR-2 were used to quantify analytes. Measured concentrations were within 10% of the certified values. Matrix spikes were performed at 10 and 50 ng/mL for the acid-digested fraction, with spike recovery ranging from 85-115% for most elements (SI Excel – Spike Recoveries).

S5 Data Processing

Field blank concentrations were used to establish the MDL. Where metals were undetectable in a field blank, the IDL was substituted. The IDL is defined as three times the standard deviation of the procedural blank divided by the slope of the calibration curve, converted to ng/m^3 for substitution purposes. The IDL was also

substituted in the event a sample/blank ratio was <1.0. The MDL was estimated to be the average of all field blanks plus 3 times the standard deviation. All samples were blank corrected by subtracting the average concentration determined in field blanks from that in the sample. Average GAPS blank concentrations (n=11) were used for GAPS samples' blank correction. Similarly, average GAPS-MC samples' blank concentrations (n=8) were used for GAPS-MC blank correction. Where metal concentrations were below the MDL, values were substituted with ½ MDL.

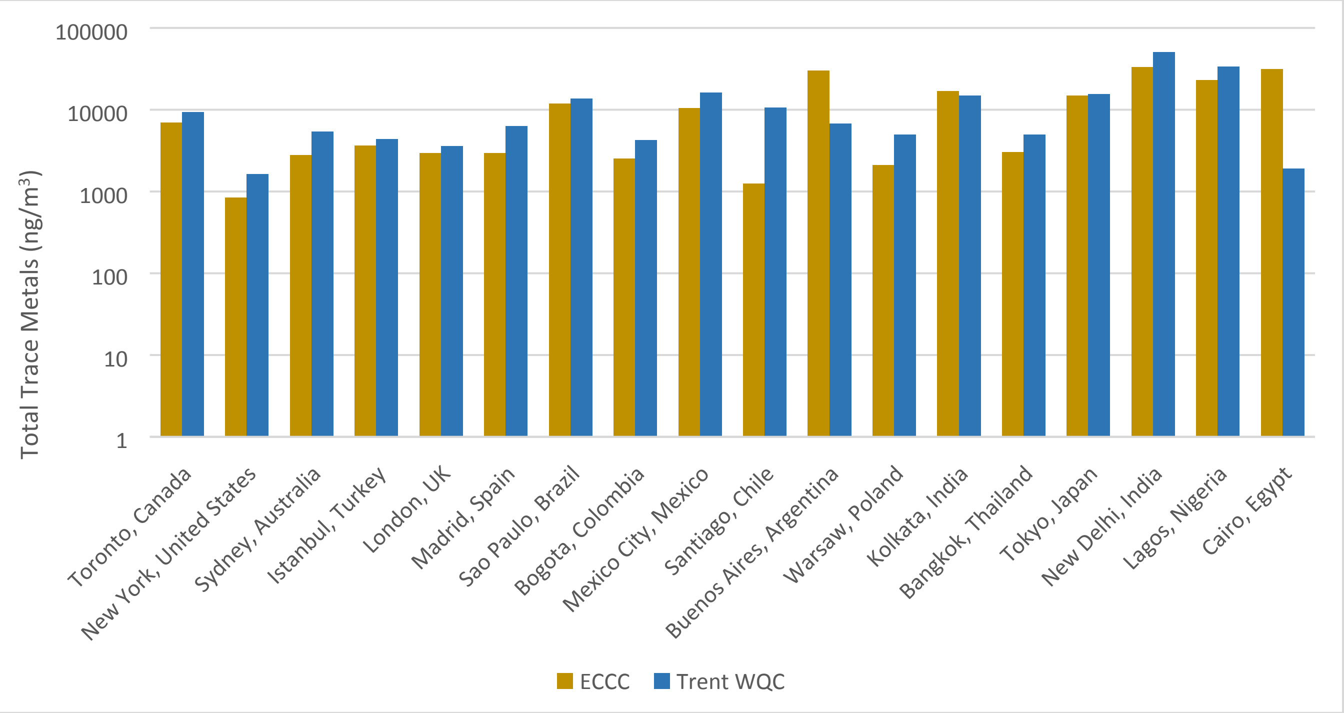

Figure S1 Acid-soluble trace metals of GAPS-MC locations as analyzed by Environment and Climate Change Canada (ECCC) and Trent University (Trent WQC).

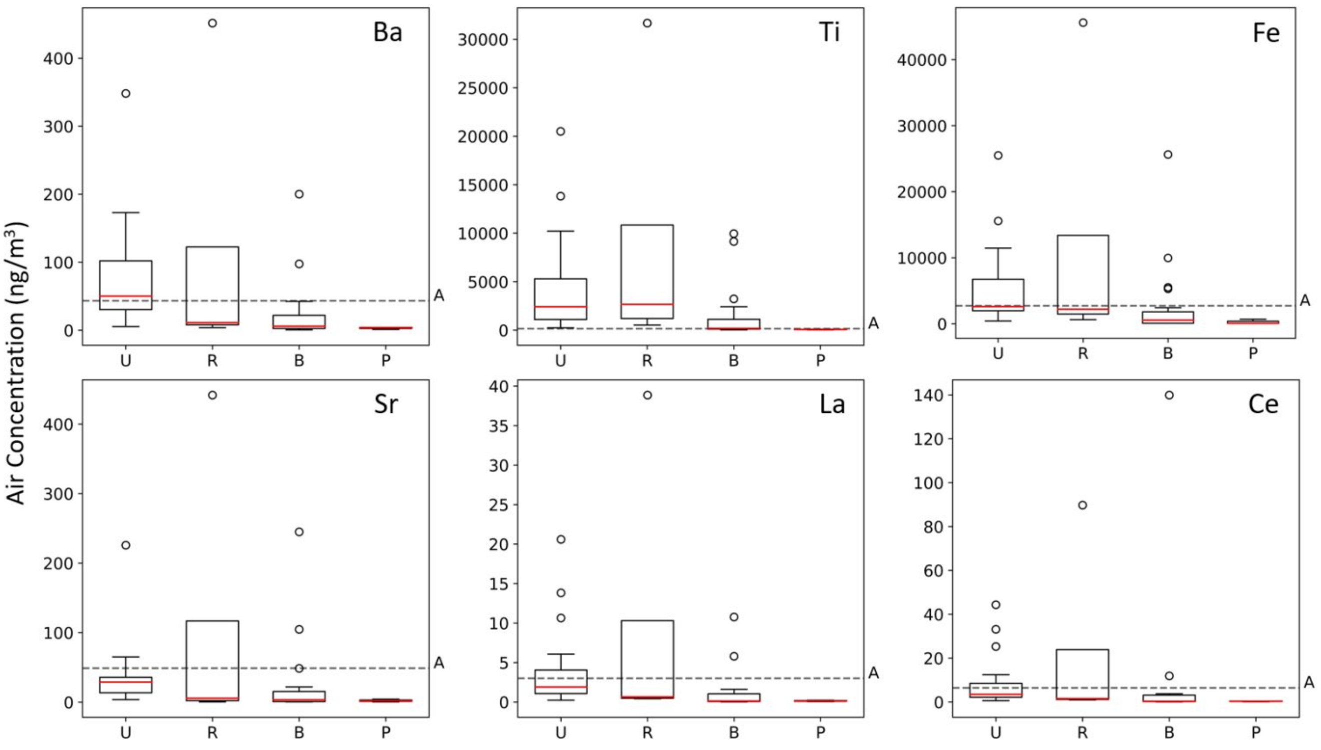

Figure S2 Global concentrations (acid-digested) of crustal metals Ba, Ti, Fe, Sr, La, Ce for GAPS and GAPS-MC 2018/2019 sampling year. The plot depicts boxplots illustrating the median, 25th and 75th percentile (whiskers marking lower/upper quartile ± IQR*1.5), and any outlying data. Concentrations are grouped by sampling site type. (Site types: U=urban, B=background, R=rural, P=polar, A=agricultural). Agricultural site concentrations marked with dashed line.

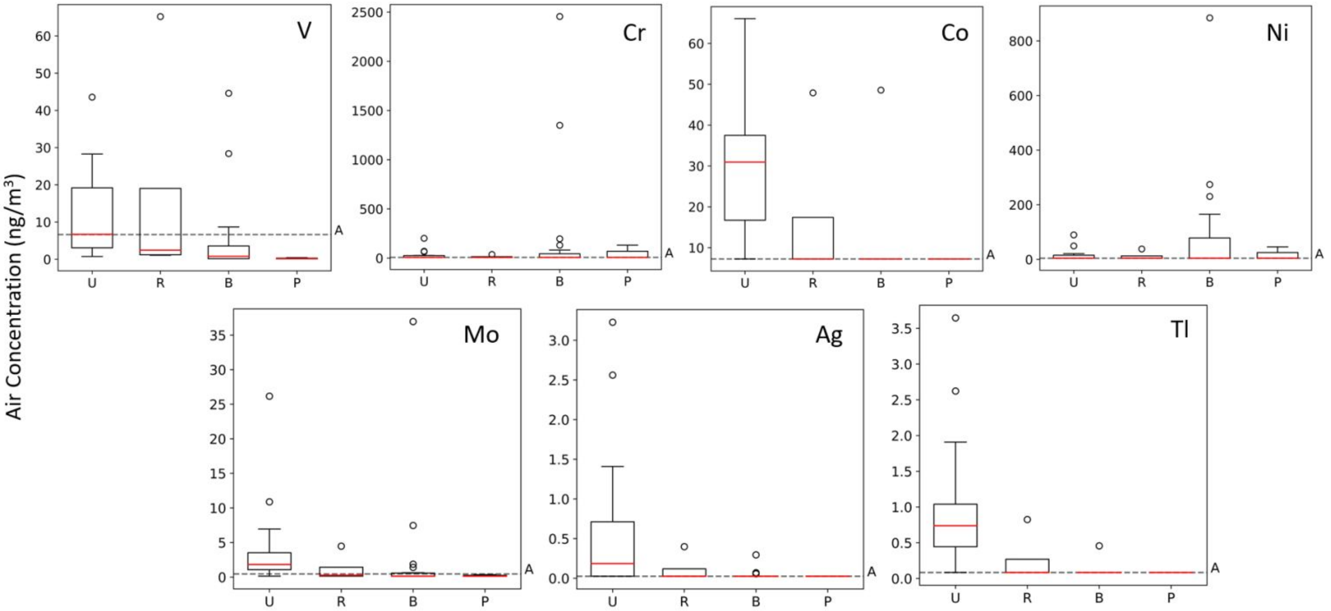

Figure S3 Global concentrations (acid-digested) of transition and post-transition metals V, Cr, Co, Ni, Mo, Ag, and Tl for GAPS and GAPS-MC 2018/2019 sampling year. The plot depicts boxplots illustrating the median, 25th and 75th percentile (whiskers marking lower/upper quartile ± IQR*1.5), and any outlying data. Concentrations are grouped by sampling site type. (Site types: U=urban, B=background, R=rural, P=polar, A=agricultural). Agricultural site concentrations marked with dashed line.

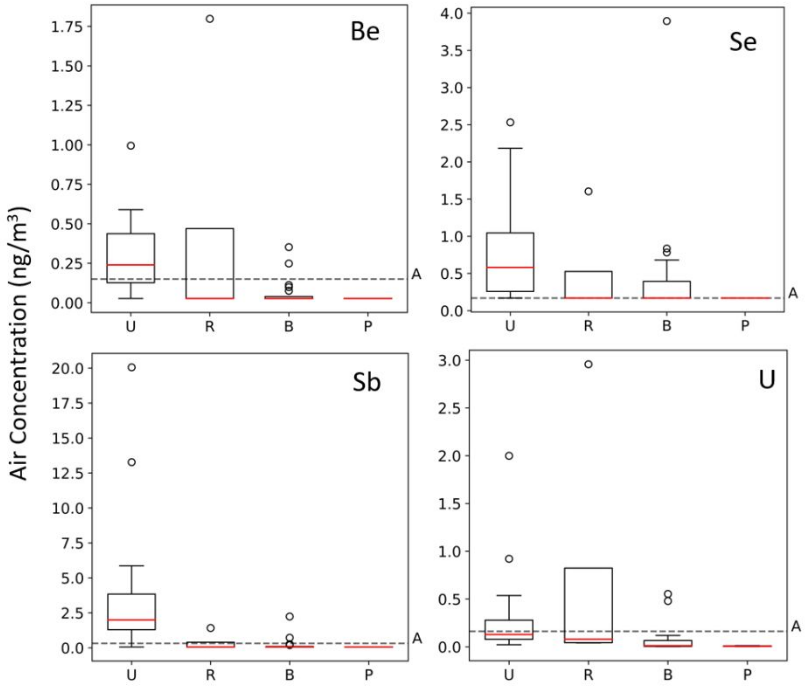

Figure S4 Global concentrations (acid-digested) of metals not classified as crustal, transition, or post-transition elements Be, Se, Sb, and U for GAPS and GAPS-MC 2018/2019 sampling year. The plot depicts boxplots illustrating the median, 25th and 75th percentile (whiskers marking lower/upper quartile ± IQR*1.5), and any outlying data. Concentrations are grouped by sampling site type. (Site types: U=urban, B=background, R=rural, P=polar, A=agricultural). Agricultural site concentrations marked with dashed line.

Table S1 Sampling site and sample deployment details.

Location	Site Code	Site Type	Latitude	Longitude	Deployment Date	Retrieval Date	Sampling Period (d)
Toronto, Canada	**MC-WE01**	**Urban**	43.6590	-79.3956	2018-07-03	2018-10-03	92
New York, USA	**MC-WE02**	**Urban**	40.7128	-74.0060	2018-07-18	2018-11-13	118
Sydney, Australia	**MC-WE03**	**Urban**	-33.8688	151.2093	2018-07-03	2018-10-03	92
Istanbul, Turkey	**MC-WE04**	**Urban**	41.0082	28.9784	2018-07-12	2018-10-22	102
London, UK	**MC-WE05**	**Urban**	51.5074	-0.1278	2018-07-12	2018-10-08	88
Madrid, Spain	**MC-WE06**	**Urban**	40.4168	-3.7038	2018-07-02	2018-10-02	92
Sao Paulo, Brazil	**MC-GR01**	**Urban**	-23.6183	-46.6355	2018-07-02	2018-10-02	92
Bogota, Colombia	**MC-GR02**	**Urban**	4.6368	-75.0834	2018-06-29	2018-10-03	96
Mexico City, Mexico	**MC-GR03**	**Urban**	19.2465	-99.1013	2018-07-02	2018-10-02	92
Santiago, Chile	**MC-GR04**	**Urban**	-33.4378	-70.6504	2019-03-01	2019-06-13	104
Buenos Aires, Argentina	**MC-GR05**	**Urban**	-34.5581	-58.4867	2019-01-17	2019-02-28	42
Warsaw, Poland	**MC-CEE01**	**Urban**	52.2298	21.0122	2018-07-03	2018-09-28	87
Kolkata, India	**MC-AS01**	**Urban**	22.5726	88.3639	2018-07-08	2018-10-22	106
Bangkok, Thailand	**MC-AS03**	**Urban**	13.7235	100.5216	2018-06-27	2018-09-27	92
Tokyo, Japan	**MC-AS04**	**Urban**	35.6895	139.6917	2018-07-09	2018-10-01	84
New Delhi, India	**MC-AS05**	**Urban**	28.5897	77.2257	2019-02-25	2019-05-17	81
Lagos, Nigeria	**MC-AF01**	**Urban**	6.5244	3.3792	2018-06-30	2018-09-30	92
Cairo, Egypt	**MC-AF03**	**Urban**	30.0141	31.4860	2018-07-01	2018-10-02	93
Alert, NU, Canada	**WE01**	**Polar**	82.4501	-63.5040	2018-12-31	2020-01-03	368
Barrow, Alaska, United States	**WE02**	**Polar**	71.3200	-156.6000	2019-01-08	2019-12-31	357
Whistler, BC, Canada	**WE06**	**Background**	50.0583	-122.9570	2018-12-31	2020-01-02	367
Ny-Ålesund, Norway	**WE13**	**Polar**	78.9072	11.8867	2019-01-08	2019-11-22	318
Malin Head, Ireland	**WE16**	**Background**	55.3717	-7.3390	2019-01-02	2020-01-02	365
Paris, France	**WE17**	**Urban**	48.8639	2.3583	2019-01-10	2020-01-10	365
Cape Grim, Australia	**WE23**	**Background**	-40.6836	144.7009	2019-01-20	2020-01-02	347
Pallas, Finland	**WE30**	**Background**	68.0000	24.2400	2018-12-31	2019-12-31	365
Fraserdale, ON, Canada	**WE32**	**Background**	49.8833	-81.5667	2019-01-04	2020-01-10	371
Ucluelet, BC, Canada	**WE33**	**Background**	48.9333	-125.5167	2018-12-19	2019-12-30	376
Point Reyes, CA, United States	**WE35**	**Background**	38.0416	-122.7944	2019-01-28	2020-01-14	351
Mauna Loa Obs, Hilo, Hawaii, USA	**WE37**	**Background**	19.5359	-155.5767	2018-12-31	2019-12-30	364
Groton, CT, USA	**WE40**	**Background**	41.3167	-72.0667	2018-01-01	2020-01-16	745
Doñana National Park, Spain	**WE41**	**Background**	37.0533	-6.5542	2018-12-17	2019-12-17	365
Longwoods, ON, Canada	**WE45**	**Rural**	42.8833	-81.4806	2018-12-31	2020-01-01	366
Arauca, Colombia	**GR04**	**Rural**	7.0128	-70.7434	2019-01-15	2020-01-20	370
Ragged Point, St. Philip, Barbados	**GR12**	**Background**	13.1651	-59.4322	2018-12-31	2020-01-14	379
Valley of the Yaqui in Sonora, Mexico	**GR16**	**Agricultural**	27.1651	-109.8405	2019-01-07	2020-01-15	373
Pierre Auger, Argentina	**GR20**	**Background**	-35.1137	-65.5999	2019-01-14	2020-01-10	361
Mendoza province, Argentina	**GR21**	**Rural**	-32.7092	-68.4004	2019-12-01	2020-01-04	34
Manizales, Colombia	**GR22**	**Background**	5.0758	-75.4367	2018-12-27	2020-01-03	372
São Jose do Maranha, Brazil	**GR23**	**Urban**	-2.5539	-44.2500	2019-01-07	2020-01-13	371
São Jose dos Ausentes, Brazil	**GR24**	**Background**	-28.5942	-49.8186	2019-01-09	2020-01-06	362

Salta, Argentina	**GR26**	**Background**	-25.0851	-66.1262	2020-02-17	2021-04-12	420
Rio Gallegos, Argentina	**GR27**	**Rural**	-51.6473	-69.2073	2020-02-27	2021-03-31	398
Concepción, Chile	**GR28**	**Urban**	-36.8293	-73.0342	2019-01-03	2020-01-03	365
Manila, Philippines	**AS11**	**Urban**	14.6519	121.0689	2019-01-07	2020-01-06	364
Bukit Kototabang, Indonesia	**AS13**	**Background**	0.2000	100.3200	2018-12-31	2020-01-06	371
Jeju Island, Korea	**AS19**	**Background**	33.2936	126.1628	2018-12-31	2019-12-31	365
Abdaly, Kuwait	**AS21**	**Background**	29.9788	47.7063	2018-12-24	2020-01-01	373
Hanimaadhoo, Maldives	**AS28**	**Background**	6.7763	73.1833	2019-03-27	2019-12-31	279
De Aar, South Africa	**AF04**	**Background**	-30.6650	23.9930	2019-04-03	2019-08-13	132
Yaba, Lagos, Nigeria	**AF10**	**Urban**	6.5177	3.3726	2018-12-31	2019-12-31	365

Table S2 Site specific sampling rates derived through the model and online tool developed by Herkert et al. (2016; 2018; The University of Iowa, 2020).

Site Name	Site Code	Average Sampling Volume per Day (m^3/d)	Sampling Period (d)	Total Volume Sampled (m^3)
Toronto, Canada	**MC-WE01**	2.049	92	189
New York, USA	**MC-WE02**	3.340	118	394
Sydney, Australia	**MC-WE03**	3.588	92	330
Istanbul, Turkey	**MC-WE04**	1.923	102	196
London, UK	**MC-WE05**	3.605	88	317
Madrid, Spain	**MC-WE06**	2.863	92	263
Sao Paulo, Brazil	**MC-GR01**	1.572	92	145
Bogota, Colombia	**MC-GR02**	2.110	96	203
Mexico City, Mexico	**MC-GR03**	1.435	92	132
Santiago, Chile	**MC-GR04**	3.132	104	326
Buenos Aires, Argentina	**MC-GR05**	2.898	42	122
Warsaw, Poland	**MC-CEE01**	2.331	87	203
Kolkata, India	**MC-AS01**	1.640	106	174
Bangkok, Thailand	**MC-AS03**	2.153	92	198
Tokyo, Japan	**MC-AS04**	2.122	84	178
New Delhi, India	**MC-AS05**	2.849	81	231
Lagos, Nigeria	**MC-AF01**	1.445	92	133
Cairo, Egypt	**MC-AF03**	1.873	93	174
Alert, NU, Canada	**WE01**	2.327	368	856
Barrow, Alaska, United States	**WE02**	4.416	357	1576
Whistler, BC, Canada	**WE06**	1.496	367	549
Ny-Ålesund, Norway	**WE13**	4.508	318	1434
Malin Head, Ireland	**WE16**	5.844	365	2133
Paris, France	**WE17**	5.553	365	2027
Cape Grim, Australia	**WE23**	5.780	347	2006
Pallas, Finland	**WE30**	5.132	365	1873
Fraserdale, ON, Canada	**WE32**	2.726	371	1011
Ucluelet, BC, Canada	**WE33**	5.106	376	1920
Point Reyes, CA, United States	**WE35**	5.507	351	1933
Mauna Loa Obs, Hilo, Hawaii, USA	**WE37**	3.889	364	1416
Groton, CT, USA	**WE40**	0.607	745	452
Doñana National Park, Spain	**WE41**	4.997	365	1824
Longwoods, ON, Canada	**WE45**	3.677	366	1346
Arauca, Colombia	**GR04**	3.478	370	1287
Ragged Point, St. Philip, Barbados	**GR12**	5.761	379	2183
Valley of the Yaqui in Sonora, Mexico	**GR16**	4.456	373	1662
Pierre Auger, Argentina	**GR20**	5.242	361	1892
Mendoza province, Argentina	**GR21**	5.853	34	199
Manizales, Colombia	**GR22**	4.253	372	1582

São Jose do Maranha, Brazil	**GR23**	0.427	371	159
São Jose dos Ausentes, Brazil	**GR24**	2.929	362	1060
Salta, Argentina	**GR26**	4.964	420	2085
Rio Gallegos, Argentina	**GR27**	6.214	398	2473
Concepción, Chile	**GR28**	5.184	365	1892
Manila, Philippines	**AS11**	3.186	364	1160
Bukit Kototabang, Indonesia	**AS13**	4.101	371	1521
Jeju Island, Korea	**AS19**	1.630	365	595
Abdaly, Kuwait	**AS21**	3.780	373	1410
Hanimaadhoo, Maldives	**AS28**	5.041	279	1406
De Aar, South Africa	**AF04**	4.818	132	636
Yaba, Lagos, Nigeria	**AF10**	1.720	365	628

Table S3 Concentrations of trace metals (ng/m^3) in air reported in selected studies from the literature. Values have been rounded to 3 significant digits where applicable. Urban studies are highlighted in grey.

	**This Study**	Sao Paulo, Brazil [1]	Kolkata, India* [2]	*Mexico City, Mexico [3]*	Bangkok, Thailand* [4]	Santiago, Chile [5]	Oxford, UK [6]	Agra, India [7]	Foshan, China [8]	Delhi, India [9]	Istanbul, Turkey [10]
Sampling Method	Passive	Active, Hi-vol	Active, Hi-vol	Active, Hi-vol	Active, Hi-vol	Active, Hi-vol	Active, Hi-vol	Active, Hi-vol	Active, Hi-vol	Active, Hi-vol	Active, Hi-vol
Sampling Period	2018-2019	2003	2003-2004	2006	2006-2007	2006-2010	2007-2008	2007-2009	2008	2008-2009	2008-2010
Metal Analysis Method	ICP-MS	ICP-MS	ICP-AES	ICP-AES, ICP-MS	ICP-AES	ICP-AES	ICP-MS	AAS	ICP-MS	FAAS	GF-AAS
Fraction	$PM_{<5}$	PM_{10}	PM_{10}	PM_{10}	TSP	PM_{10}	$PM_{<2.5}$	$PM_{2.5}$	$PM_{2.5}$	PM_{10}	TSP
Al	1110	1570			3463.6-3510.3	1869-4319					
Ti	41.3	570		81		19-294					
V	3.15	10		25		6.-19	0.05-8.6		40		1.2-3.1
Cr	6.73	10	6.3-7.1	4	9.5-12.0	9.-19	0.02-0.76	600		130-230	3.8-7.9
Mn	45.2	30	2.0-2.1	32	49.0-81.7	78-123	0.71-12.4	100	200.6	250-320	7.6-16.7
Fe	1910	1860	87.2-123.2		1527.8-5578.6	2012-4784	0.56-22.4	1900		8000-11200	486.2-1303.4
Co	7.24	10		1		1.-2	0.001-0.10				
Ni	4.17	10	6.8-8.3	5	461.9-929.4	1.-14	0.02-3.6	200		280-370	2.9-4.8
Cu	12.9	80		110	227.5-263.1	66-199	0.45-16.3	200	283.8	180-270	2.2-19.1
Zn	75.8	240	489.5-534.5	482	2186.7-2463.9	123-895	0.20-4.77		221.4	4100-4700	
Sr	13.2			16							
As	0.57			6		12-43			76.6		
Se	0.17					3.-8					
Mo	0.74						0.001-0.12				
Cd	0.08	10	2.2-5.2	3	1.6-2.7	1.-2	0.006-0.54	500	42.6	10.-20	0.4-0.9
Sb	0.32					9.-23					
Ba	22.9					55-178					
La	0.91										
Ce	2.06										
Tl	0.08										
Pb	5.23	10	40.4-118.6	111	53.5-197.4	42-125	0.21-17.6	400	675.7	270-460	4.8-16.7

[1] Vasconcellos et al., 2007, [2] Karar & Gupta, 2006, [3] Querol et al., 2008, [4] Rungratanaubon et al., 2008, [5] Rubio et al., 2018, [6] Witt et al., 2010, [7] Massey et al., 2013, [8] Tan et al., 2014, [9] Khillare & Sarkar, 2012, [10] Sahin et al., 2013
Bold Median values
Italics Water-soluble fraction only
*Range indicates low/high concentrations between residential and industrial sites.

Table S6 (continued)

	Cairo, Egypt [11]	Istanbul, Turkey [12]	Central Taiwan [13]	Buenos Aires, Argentina [14]	Toronto, Canada [14]	Sao Paulo, Brazil [15]	Sao Paulo, Brazil [16]	Sao Paulo, Brazil [17]	Lagos, Nigeria [18]	**Delhi, India [19]**
Sampling Method	Active, Low-vol	Active, Hi-vol	Active, Hi-vol	Active, Hi-vol	Active, Hi-vol	Active, Hi-vol	Active, Hi-vol	Active, Hi-vol	Active, Hi-vol	Active, Hi-vol
Sampling Period	2009-2010	2009-2010	2013-2014	Various, 2013-2019	Various, 2013-2019	2014	2014-2015	2015-2016	2017	2018-2019
Metal Analysis Method	AAS	EDXRF	ICP-MS	ICP-MS	ICP-MS	ICP-MS	EDXRF	ICP-MS	EDXRF	EDXRF
Fraction	SPM	$PM_{1-2.5}$	$PM_{2.5,\ 10}$	$PM_{2.5}$	$PM_{2.5}$	$PM_{2.5,\ 10}$	$PM_{2.5}$	$PM_{2.5}$	$PM_{2.5}$	$PM_{2.5-10}$
Al		393-602	1010-1490			981	315	1006		199
Ti		13.9-168	18-55.5	1.7	0.83		30		18.5-22.7	30.5
V		0.22-2.54	7.78-10.7	2.48	0.09		2		6.0-9.9	0.78
Cr	25-46	5.87-122	26.1-30.7	1.1	0.72	20	4		1.6	3.5
Mn	140-204	1.85-42.1	12.4-20.1	2.52	1.59	33	11		3.9-6.2	20.8
Fe	3000-4400	27.5-117.3	171-571	94	46.5	1269	450		109.5-196.9	500
Co	21-40	0.41-0.52	0.263-0.531			0.59		0.08	6.7-20.1	<DL
Ni	41-68		5.73-9.84			6.6	17	39.5	25.4-67.2	1.2
Cu	51-84	3.66-19.6	9.11-15.7	5.17	2.71	188	32		9.5-47.5	27.4
Zn	115-167	108-385	102.9-131	20.8	10.7	193	320		11.3-17.5	445
Sr		10.0-10.8	1.99-5.03			4.8				<DL
As		0.69-0.75	3.35-3.39	0.43	0.31	2.2		1.3		10
Se				0.36	0.29	2.6	24		24.14	2.8
Mo		0.09-1.08	1.52-1.55							
Cd	20-48		0.704-0.787	0.3	0.04	1.2	8	0.6		<DL
Sb			2.65-2.99							<DL
Ba		21.1-242	8.12-12.3	5.08	3.82					4.8
La			0.201-0.591							
Ce			0.239-0.806							
Tl						0.15				<DL
Pb	450-650		21.2-23.1	10.47	1.38	42	44			181

[11] Hassan et al., 2013, [12] Onat et al., 2013, [13] Hsu et al., 2016, [14] McNeill et al., 2020, [15] Pereira et al., 2017, [16] de Miranda et al., 2018, [17] de Oliveira Alves et al., 2020, [18] Alani et al., 2019, [19] Rai et al., 2020
Bold Median values

Table S4 Average $PM_{2.5}$ and PM_{10} concentrations at GAPS-MC locations during sampling. Concentrations derived from average AQI index at each location. AQI converted to PM concentration (µg/m^3) using AirNow AQI Calculator (https://www.airnow.gov/aqi/aqi-calculator/).

Location	PM2.5	PM10	Source	Website
**Toronto, Canada**	9.6		Air Quality Ontario - the Ontario Ministry of the Environment and Climate Change	https://waqi.info/
**New York, USA**	7.9	17	New York State Department of Environmental Conservation (NYSDEC) - Air Now - US EPA (PM2.5); https://www.epa.gov/outdoor-air-quality-data/download-daily-data (PM10)	https://waqi.info/
**Sydney, Australia**	8.1	17	Office of Environment and Heritage - NSW	https://waqi.info/
**Istanbul, Turkey**	15.9	38	Turkey National Air Quality Monitoring Network (Ulusal Hava Kalitesi İzleme Ağı)	https://waqi.info/
**London, UK**	16.3	25	UK-AIR, air quality information resource - Defra, UK - London Air Quality Network - Environmental Research Group, King's College London	https://waqi.info/
**Madrid, Spain**	22	40	Calidad del Air en Madrid, Servicio de Protección de la Atmósfera - European Environment Agency	https://waqi.info/
**Sau Paulo, Brazil**	20.6	33	CETESB - Companhia Ambiental do Estado de São Paulo	https://waqi.info/
**Bogota, Colombia**	8.3	17	OAB - El Observatorio Ambiental de Bogotá	https://waqi.info/
**Mexico city, Mexico**	18.2	29	SINAICA - Sistema Nacional de Información de la Calidad del Aire en Mexico - INECC - Instituto Nacional de Ecología y Cambio Climático	https://waqi.info/
**Santiago, Chile**	27.3	92	Sistema Nacional de Calidad del Aire en Chile	https://waqi.info/
**Buenos Aires, Argentina**	11.2	33	Control y Monitoreo de la Autoridad de Cuenca Matanza Riachuelo (ACUMAR)	https://waqi.info/
**Warsaw, Poland**	17.8	34	Regional Inspectorate for Environmental Protection in Warsaw (Wojewódzki Inspektorat Ochrony Środowiska w Warszawie) - Główny inspektorat ochrony środowiska	https://waqi.info/
**Kolkata, India**	27.7	60	CPCB - India Central Pollution Control Board	https://waqi.info/
**Bangkok, Thailand**	18.2	36	World Meteorological Organization - surface synoptic observations (WMO-SYNOP) - Division of Air Quality Data, Air Quality and Noise Management Bureau, Pollution Control Department.	https://waqi.info/
**Tokyo, Japan**	10.5	21	Japan Atmospheric Environmental Regional Observation System (環境省大気汚染物質広域監視システム) - Tokyo, Japan Environment Agency (東京都環境局)	https://waqi.info/
**New Delhi, India**	55.4	251	Delhi Pollution Control Commitee (Government of NCT of Delhi) - CPCB - India Central Pollution Control Board	https://waqi.info/
**Cairo, Egypt**		228	Egyptian Environment Affairs agency, Air quality report of 2018	

References

Alani, R.A., Ayejuyo, O.O., Akinrinade, O.E., Badmus, G.O., Festus, C.J., Ogunnaike, B.A., Alo, B.I. The level $PM_{2.5}$ and the elemental compositions of some potential receptor locations in Lagos, Nigeria. Air Quality, Atmos. Health. 2019, 12, 1251-1258.

de Miranda, R.M., de Fatima Andrade, M., Ribeiro, F.N.D., Francisco, K.J.M., Pérez-Martínez, P.J. Source apportionment of fine particulate matter by positive matrix factorization in the metropolitan area of São Paulo, Brazil. J. Clean. Prod. 2018, 202, 253-263.

de Oliveira Alves, N., Pereira, G.M., Di Domenico, M., Costanzo, G., Benevuto, S., de Oliveira Fonoff, A.M., de Souza Xavier Costa, N., Júnior, G.R., Kajitani, G.S., Moreno, N.C., Fotoran, W., Torres, J.I., de Andrade, J.B., Veras, M.M., Artaxo, P., Frederico, C., Menck, M., de Castro Vasconcellos, P., Saldiva, P. Inflammation response, oxidative stress and DNA damage caused by urban air pollution exposure increase in the lack of DNA repair XPC protein. Environ. Int. 2020, 145, 106150.

Hassan, S.K., El-Abssawy, A.A., Abdel-Maksoud, A.S., Abdou, M.H., Khoder, M.I. Seasonal Behaviours and Weekdays/Weekends Differences in Elemental Composition of Atmospheric Aerosols in Cairo, Egypt. Aero. Air Qual. Res. 2023, 13, 1552-1562.

Herkert, N.J., Martinez, A., Hornbuckle, K.C. A Model Using Local Weather Data to Determine the Effective Sampling Volume for PCB Congeners Collected on Passive Air Samplers. Environ. Sci. Technol. 2016, 50 (13), 6690-6697.

Herkert, N.J., Spak, S.N., Smith, A., Schuster, J.K., Harner, T., Martinez, A., Hornbuckle, K.C. Calibration and evaluation of PUF-PAS sampling rates across the Global Atmospheric Passive Sampling (GAPS) network. Environ. Sci. : Processes & Impacts. 2018, 20, 210-219.

Hsu, C-Y., Chiang, H-C., Lin, S-L., Chen, M-J., Lin, T-Y., Chen, Y-C. Elemental characterization and source apportionment of PM_{10} and $PM_{2.5}$ in the western coastal area of central Taiwan. Sci. Tot. Environ. 2016, 541, 1139-1150.

Karar, K., Gupta, A.K. Seasonal variations and chemical characterization of ambient PM_{10} at residential and industrial sites of an urban region of Kolkata (Calcutta), India. Atmos. Res. 2006, 81, 36-53.

Khillare, P.S., Sarkar, S. Airborne inhalable metals in residential areas of Delhi, India: distribution, source apportionment and health risks. Atmos. Pollut. Res. 2012, 3, 46-54.

Massey, D.D., Kulshrestha, A., Taneja, A. Particulate matter concentrations and their related metal toxicity in rural residential environment of semi-arid region of India. Atmos. Environ. 2013, 67, 278-286.

McNeill, J., Snider, G., Weage, C.L., Walsh, B., Bissonnette, P., Stone, E., Abboud, I., Akoshile, C., Anh, N.X., Balasubrananian, R., Brook, J., Coburn, C., Cohen, A., Dong, J., Gagnon, G., et al. Large global variations in measured airborne metal concentrations driven by anthropogenic sources. Scientific Reports. 2020, 10, 21817.

Onat, B., Sahin, U.A., Akyuz, T. Elemental characterization of $PM_{2.5}$ and PM_1 in dense traffic area in Istanbul, Turkey. Atmos. Pollut. Res. 2013, 4, 101-105.

Pereira, G.M., Teinilä, K., Custódio, D., Santos, A.G., Xian, H., Hillamo, R., Alves, C.A., de Andrade, J.B., da Rocha, G.O., Kumar, P., Balasubrananian, R., de Fátima Andrade, M., de Castro Vasconcellos, P. Particulate pollutants in the Brazilian city of São Paulo: 1-year investigation for the chemical composition and source apportionment. Atmos. Chem. Phys. 2017, 17, 11943-11969.

Querol, X., Pey, J., Minguillon, M.C., Pérez, N., Alastuey, A., Viana, M., Moreno, T., Bernabé, R.M., Blanco, S., Cárdenas, B., Vega, E., Sosa, G., Escalona, S., Ruiz, H., Artíñano, B. PM speciation and sources in Mexico during the MILAGRO-2006 Campaign. Atmos. Chem. Phys. 2008, 8, 111-128.

Rai, P., Furger, M., Haddad, I.E., Kumar, V., Wang, L., Singh, A., Dixit, K., Bhattu, D., Petit, J-E., Ganguly, D., Rastogi, N., Baltensperger, U., Tripathi, S.N., Slowik, J.G., Prévôt, A.S.H. Real-time measurement and source apportionment of elements in Delhi’s atmosphere. Sci. Tot. Environ. 2020, 742, 140332.

Rubio, M.A., Sánchez, K., Richter, P., Pey, J., Gramsch, E. Partitioning of the water soluble fraction of trace elements in the city of Santiago, Chile. Atmósfera. 2018, 31, 373-387.

Rungratanaubon, T., Wangwongwatana, S., Panich, N. Characterization and Source Identification of Trace Metals in Airborne Particulates of Bangkok, Thailand. Ann. N.Y. Acad. Sci. 2008, 1140, 297-307.

Sahin, Ü.A., Onat, B., Polat, G. Trace element concentrations of size-fractionated particulate matter in the atmosphere of Istanbul, Turkey. Air Pollut. XXI. 2013, 174, 137-147.

Tan, J-H., Duan, J-C., Ma, Y-L., Yang, F-M., Cheng, Y., He, K-B., Yu, Y-C., Wang, J-W. Source of atmospheric heavy metals in winter in Foshan, China. Sci. Tot. Environ. 2014, 493, 262-270.

The University of Iowa. PUF-PAS Sampling Rate Model Interface. www.pufpasvolume.org (April 2023).

Vasconcellos, P.C., Balasubramanian, R., Bruns, R.E., Sanchez-Ccoyllo, O., Andrade, M.F., Flues, M. Water-Soluble Ions and Trace Metals in Airborne Particles Over Urban Areas of the State of São Paulo, Brazil: Influences of Local Sources and Long Range Transport. Water Air Soil Pollut. 2007, 186, 63-73.

Witt, M.L.I., Meheran, N., Mather, T.A., de Hoog, J.C.M., Pyle, D.M. Aerosol trace metals, particle morphology and total gaseous mercury in the atmosphere of Oxford, UK. Atmos. Environ. 2010, 44, 1524-1538.
